# Fatty Acid-Binding Protein 3 Expression in the Brain and Skin in Human Synucleinopathies

**DOI:** 10.3389/fnagi.2021.648982

**Published:** 2021-03-25

**Authors:** Hideki Oizumi, Kenshi Yamasaki, Hiroyoshi Suzuki, Takafumi Hasegawa, Yoko Sugimura, Toru Baba, Kohji Fukunaga, Atsushi Takeda

**Affiliations:** ^1^Department of Neurology, National Hospital Organization, Sendai Nishitaga Hospital, Sendai, Japan; ^2^Department of Dermatology, Graduate School of Medicine, Tohoku University, Sendai, Japan; ^3^Department of Clinical Pathology, National Hospital Organization, Sendai Medical Center, Sendai, Japan; ^4^Department of Neurology, Graduate School of Medicine, Tohoku University, Sendai, Japan; ^5^Department of Pharmacology, Graduate School of Pharmaceutical Sciences, Tohoku University, Sendai, Japan; ^6^Department of Cognitive and Motor Aging, Graduate School of Medicine, Tohoku University, Sendai, Japan

**Keywords:** α-synuclein, multiple system atrophy, fatty acid-binding protein, human, Parkinson’s disease

## Abstract

Parkinson’s disease (PD) and multiple system atrophy are types of adult-onset neurodegenerative disorders named synucleinopathies, which are characterized by prominent intracellular α-synuclein (αSyn) aggregates. We have previously found that αSyn aggregates and the vulnerability of dopaminergic neurons in the mouse brain are partly associated with the expression of fatty acid-binding protein 3 (FABP3, heart FABP). However, it remains to be elucidated whether FABP3 accumulation is associated with αSyn aggregates in human tissues. Here, we histologically studied FABP3 expression in human tissues obtained from patients with synucleinopathies, patients with Alzheimer disease (AD) and controls. We found that (1) a variety of neurons expressed the FABP3 protein in human brain tissues, (2) FABP3 was colocalized with αSyn aggregates in the brains of individuals with synucleinopathies but not with amyloid β or p-tau aggregates in the brains of individuals with AD, and (3) FABP3 was not present in p-αSyn deposits in biopsied skin tissues from individuals with PD. These findings suggest that FABP3 expression is associated with αSyn aggregation in synucleinopathies and provide new insights into the involvement of FABP3 in synucleinopathies.

## Introduction

Parkinson’s disease (PD) is a secondary common neurodegenerative disorder affecting >1% of the population over 65 years of age worldwide (de Lau and Breteler, 2006). The histopathological features of PD include loss of dopaminergic (DA) neurons in the substantia nigra (SN) and the presence of cytoplasmic protein aggregates, known as Lewy bodies (LBs) (Gibb and Lees, 1988). α-Synuclein (αSyn), a 140-amino acid protein, is associated with synaptic vesicles in presynaptic nerve terminals, and β-sheet fibrillar αSyn aggregates are major components of LBs (Spillantini et al., 1998). αSyn aggregation is associated with progressive loss of DA neurons, implicating αSyn in PD pathogenesis. In addition, duplication/triplication and missense mutations (A53T, and A30P, etc.) in the αSyn gene *SNCA* are linked to familial early-onset PD (Polymeropoulos et al., 1997; Kruger et al., 1998; Singleton et al., 2003; Chartier-Harlin et al., 2004; Farrer et al., 2004; Zarranz et al., 2004). Therefore, PD and dementia with LBs (DLB) are designated as synucleinopathies, which are neurodegenerative disorders characterized by prominent intracellular αSyn aggregates (McCann et al., 2014). Multiple system atrophy (MSA) is an adult-onset neurodegenerative disorder that is clinically characterized by a combination of poorly levodopa (L-dopa)-responsive parkinsonism, cerebellar dysfunction and autonomic failure (Yabe et al., 2006). The histopathological features of MSA include the presence of glial and neuronal protein aggregates, known as glial cytoplasmic inclusions (GCIs) and neuronal cytoplasmic inclusions (NCIs), respectively (Wakabayashi et al., 1998; Wakabayashi and Takahashi, 2006; Homma et al., 2016). Similar to LBs, GCIs and NCIs are composed largely of αSyn aggregates. Thus, on the basis of histopathological observations, MSA is also classified as a synucleinopathy (McCann et al., 2014). However, the molecular mechanisms of αSyn aggregation remain to be elucidated.

There is increasing evidence that lipid metabolism plays crucial roles in the pathogenesis of synucleinopathies (Oueslati, 2016; Xicoy et al., 2019). In particular, fatty acid (FA) metabolism is noted to occur in αSyn aggregates (Mollenhauer et al., 2007a; Wada-Isoe et al., 2008a; Paisan-Ruiz et al., 2009; Yoshino et al., 2010; Tomiyama et al., 2011; Sumi-Akamaru et al., 2016). FA-binding proteins (FABPs) are lipid chaperones that mediate biological processes and systemic metabolic homeostasis through regulation of diverse lipid signals. Among FABPs, FABP3 is expressed mainly in the heart but is distributed in the kidneys, skeletal muscle, aorta, lungs, mammary glands, placenta, testes, ovaries, adrenal grands, stomach and brain (Furuhashi and Hotamisligil, 2008; Schulz-Schaeffer, 2010). Interestingly, in a previous study on model mice with PD induced by 1-methyl-1,2,3,6-tetrahydropyridine (MPTP), we found that αSyn binds to FABP3 and that αSyn aggregates with FABP3 accumulation are abundant in damaged DA neurons (Shioda et al., 2014). We also previously found that FABP3-knockout mice are resistant to MPTP-induced DA neurodegeneration in the SN and to MPTP-induced motor dysfunction (Shioda et al., 2014). Consistent with this finding, MPTP-induced αSyn aggregation in DA neurons is attenuated in FABP3-knockout mice (Shioda et al., 2014). Thus, FABP3 expression seems to be involved in the pathogeneses of αSyn aggregation and DA neuron degeneration in PD model mice. Serum FABP3 is a potential diagnostic marker for PD and DLB because the levels of this protein are higher in PD and DLB patients than in AD patients (Mollenhauer et al., 2007a; Wada-Isoe et al., 2008a). It has also been reported that elevated FABP3 levels in cerebrospinal fluid (CSF) are associated with future dementia in individuals with PD (Backstrom et al., 2015).

Although FABP3 has been suggested to promote αSyn aggregation in animal models and to potentially be a useful biomarker for synucleinopathies, it remains to be elucidated whether FABP3 accumulation is associated with αSyn aggregation in human tissues. Here, we pathologically studied FABP3 expression in human tissues obtained from patients with synucleinopathies, patients with Alzheimer disease (AD) and controls (CNs).

## Materials and Methods

### Human Tissue Sources

Brain tissues from autopsies: We examined consecutive adult autopsy cases that had been registered at the National Hospital Organization (NHO) Sendai Medical Center. The ages of the subjects ranged from 58 to 89 years, with an average of 73.7 years. The 16 cases comprised eight female and eight male patients. The neuropathological diagnoses included four cases of PD, six cases of MSA, four CNs, and two cases of AD. None of the patients had kindred relationships with each other. The whole brains were fixed in 20% buffered formalin (Wako, Osaka, Japan) for 5–11 days. Serial coronal sections were dehydrated in a graded ethanol series, cleared in xylene, and embedded in paraffin using an automated tissue processor.

Biopsied skin tissues: Previous reports have shown that the presence of dermal αSyn deposits in proximal body sites is a sensitive biomarker for PD diagnosis, helping to differentiate PD from other parkinsonisms (Ikemura et al., 2008; Donadio et al., 2014; Zange et al., 2015). We recruited 10 PD patients fulfilling the diagnostic criteria of the UK PD brain bank (Gibb and Lees, 1988) for skin biopsy. All patients with PD showed late-onset disorder (at >45 years of age), were treated with L-dopa only or in combination with other medications and had well-controlled motor symptoms. None of the patients had kindred relationships with each other. In this study, 3-mm punch biopsies were taken from proximal body sites. Skin specimens were collected from the cervical C8 paravertebral area, i.e., close to the spinal ganglia (Donadio et al., 2014) and from an axilla. Skin biopsy was performed by using a 3-mm disposable punch with sterile technique after topical anesthesia with lidocaine. No suturing was required. We performed fixation in 4% paraformaldehyde for 24 h by using a previously described method (Ikemura et al., 2008), since this type of fixation increases immunohistochemical sensitivity for LB and Lewy neurite detection. Serial sections were dehydrated in a graded ethanol series, cleared in xylene, and embedded in paraffin using an automated tissue processor.

The procedures used were approved by the local human ethics committee and followed the Declaration of Helsinki regarding international clinical research involving humans. All subjects gave written informed consent to participate in the study.

### Histological Immunofluorescence (IF) Staining

To enhance LB labeling, we pretreated all samples with formic acid (Sengoku et al., 2008). The sections were double-immunostained overnight with primary antibodies. The following antibodies were used in this study: rabbit polyclonal anti-FABP3 antibodies (1:100 dilution, Proteintech, Chicago, IL, United States), a mouse monoclonal p-αSyn (Ser 129) antibody (1:1,000 dilution, Wako, Richmond, VA, United States), a mouse monoclonal anti-αSyn aggregate antibody (BioLegend, San Diego, CA, United States), a mouse monoclonal anti-amyloid β antibody (1:100 dilution, Abcam, ab11132, Cambridge, MA, United States) and a mouse monoclonal anti-p-tau antibody (1:100 dilution, Innogenetics, Ghent, Belgium). The sections were then washed, and secondary antibodies were added for a 2-h incubation. The secondary antibodies, anti-mouse Alexa Fluor 488, anti-rabbit Alexa Fluor 594, anti-mouse Alexa Fluor 594, and anti-rabbit Alexa Fluor 488, were obtained from Jackson ImmunoResearch (West Grove, PA, United States) and were used at a 1:500 dilution. Then, a TrueBlack lipofuscin autofluorescence quencher (Biotium Inc. Fremont, CA, United States) was used to remove artifacts caused by lipofuscin in these tissues. The sections were viewed and analyzed under a microscope system (BZ-X700, Keyence, Osaka, Japan). The oculomotor nucleus were identified by referring to the hematoxylin and eosin staining of the adjacent sections used for fluorescence staining.

### Immunohistochemistry

We pretreated all samples with formic acid. The following primary antibodies were used: a mouse monoclonal anti-p-tau antibody (1:100 dilution, Innogenetics, Ghent, Belgium). The signals from monoclonal and polyclonal antibodies were detected by using the automatic system on a VENTANA NX20 with the I-View DAB Universal Kit (Roche, Basel, Switzerland) according to the manufacturer’s instructions. Sections were counter-stained with hematoxylin.

### Semiquantitative Analysis of IF Staining

For semiquantitative analysis of p-αSyn and αSyn fibril protein aggregation with FABP3 accumulation, double-positive cells were analyzed via immunohistochemical grading of double-positive aggregates in a 20× field according to the following scoring system; +++, more than 5 intracellular aggregates in a field; ++, 2–5 aggregates in a field; +, one aggregate in a field; and –, no aggregates. For semiquantitative analysis of p-αSyn deposits and FABP3 accumulations in biopsied skin tissues, positive areas were analyzed via immunohistochemical grading of double-positive deposits in a 20× field according to the following scoring system; ++, more than 30 deposits in a field; +, 1–29 deposits in a field; and –, no deposit. We also examined the ratio of FABP3-immunoreactive (ir) deposits to anti-p-αSyn or anti-αSyn fibril-ir aggregates. These ratios were measured by the software programs Hybrid Cell Count and Macro Cell Count (Keyence, Osaka, Japan) in 20×-magnified fields for brain tissues [the striatum for MSA and the entorhinal cortex (EC) for PD]. We quantify the luminance of the green-labeled aggregates and the luminance of the red-labeled target proteins expressed. The ratio was then calculated using the luminance of the green aggregates as the denominator and the red target protein as the numerator.

### Statistical Evaluation

All values are expressed as the means ± SEs. Differences between groups were examined for statistical significance using independent *t*-tests. The data were analyzed with IBM Statistics software (version 25). A *P* value <0.01 was considered to indicate a statistically significant difference.

## Results

### FABP3 Is Expressed in Neurons in Normal Brain Tissues

**FIGURE 1:**
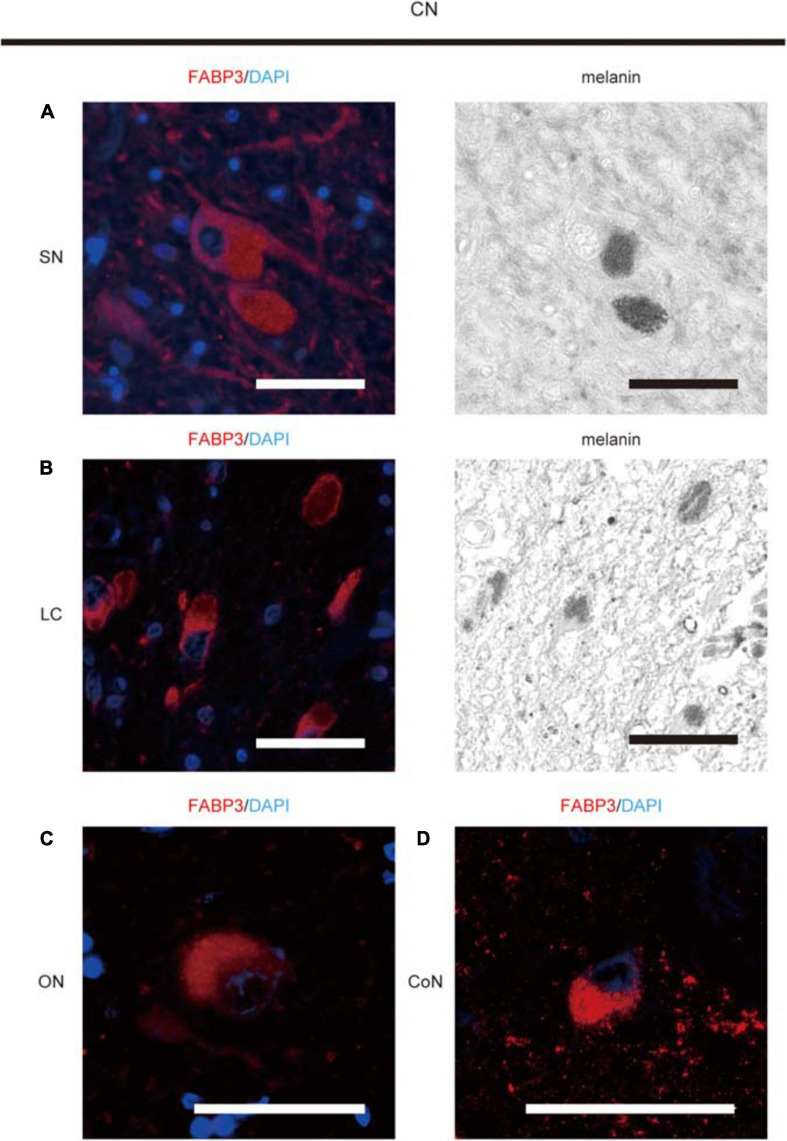
Immunohistochemical analysis of FABP3 protein expression in autopsied brain tissues of CNs. We used fluorescence microscopy to show FABP3 expression and optical microscopy to identify melanin-positive DA neurons and locus coeruleus (LC) neurons. The nuclei of neurons were identified morphologically by DAPI (blue) staining. IF staining of FABP3 (red) and melanin (black) in DA neurons in the SN of CNs is shown in high-magnification images **(A)**. IF staining of FABP3 (red) and melanin (black) in LC neurons in the pons of CNs is shown in high-magnification images **(B)**. IF staining of FABP3 (red) in the oculomotor neurons (ON) of CNs is shown in high-magnification images **(C)**. IF staining of FABP3 (red) in the cortical neurons (CoN) in the EC of CNs is shown in high-magnification images **(D)**. FABP3-ir proteins in melanin-positive neurons were expressed mostly in the cytoplasm. FABP3-ir proteins were also expressed in oculomotor neurons and cortical neurons other than melanin-positive neurons. Scale bars = 50 μm.

FABP3-ir proteins were expressed in melanin-positive DA neurons in the SN (Figure 1A), in melanin-positive locus coeruleus neurons in the pons (Figure 1B), in oculomotor neurons in the midbrain (Figure 1C) and cortical neurons in the EC (Figure 1D) in autopsied brain tissues from CNs. FABP3-ir proteins in melanin-positive neurons were expressed mostly in the cytoplasm. FABP3 proteins were also expressed in oculomotor neurons and cortical neurons other than melanin-positive neurons.

### FABP3 Protein Expression Is Associated With α Syn Aggregates in Alpha Synucleinopathies

**FIGURE 2:**
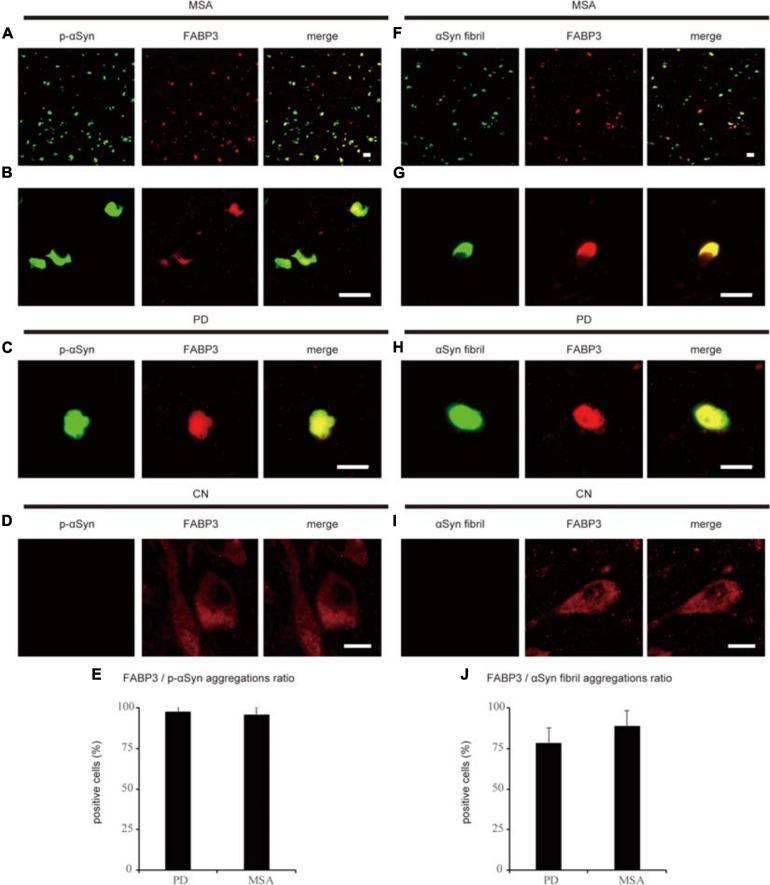
Correlation between FABP3 protein accumulation and αSyn aggregation in synucleinopathies. IF staining of FABP3 (red) and p-αSyn (green) in the SN in MSA tissue is shown in low-magnification **(A)** and high-magnification **(B)** images; staining in the SN in PD tissue is shown in low-magnification images **(C)**; and staining in the SN in CN tissue is shown in high-magnification images **(D)**. **(A–C)** shows that FABP3 was colocalized with p-αSyn aggregates in synucleinopathies. In contrast, **(D)** shows that p-αSyn aggregates were not observed in the SN in CNs, although FABP3 protein was detected in DA neurons. The ratio of FABP3 protein accumulations to p-αSyn aggregates in the striatum in MSA tissue and in the EC in PD tissue was quantitatively analyzed **(E)** (*p* = 0.666). **(E)** shows that FABP3 accumulations were colocalized with most p-αSyn aggregates in both PD and MSA tissues. IF staining of FABP3 (red) and αSyn fibrils (green) in the SN in MSA tissue is shown in low-magnification **(F)** and high-magnification **(G)** images; staining in the SN in PD tissue is shown in low-magnification images **(H)**; and staining in the SN in CN tissue is shown in high-magnification images **(I)**. **(F–H)** shows that FABP3 accumulations were colocalized with αSyn fibrils in synucleinopathies. In contrast, **(I)** shows that αSyn fibrils were not observed in the SN in CNs, although FABP3 protein was detected in DA neurons. The ratio of FABP3 protein accumulations to αSyn fibrils in the striatum in MSA tissue and in the EC in PD tissue was quantitatively analyzed **(J)** (*p* = 0.119). **(J)** shows that FABP3 accumulations were colocalized with approximately 80% of αSyn fibrils in both PD and MSA tissues. Scale bars = 20 μm.

We next examined whether phosphorylated αSyn (p-αSyn) colocalized with FABP3 in synucleinopathies. As shown in Table 1, double-positive (p-αSyn-ir and FABP3-ir) aggregates were observed in the SN and EC in all four PD cases and in the striatum in all six MSA cases but not in the SN or EC in any of the four CNs. Figures 2A–E shows images of IF staining of FABP3 (red) and p-αSyn aggregates (green) in the striatum in MSA tissues (Figure 2A, low magnification; 2B, high magnification) and in the EC in PD tissues (Figure 2C, low magnification). There were no significant differences in the ratio of FABP3 accumulations to p-αSyn aggregates between PD and MSA tissues (Figure 2E) (*t* = 0.448, *p* = 0.666). FABP3 accumulations were colocalized with most p-αSyn aggregates in both PD and MSA tissues (Figure 2E). In contrast, p-αSyn aggregates were not observed in the SN in CNs, although FABP3 was detected in DA neurons (Figure 2D).

**TABLE 1:** Demographic profiles of autopsy cases and the results of immunostaining for colocalization of p-αSyn and αSyn fibril aggregates with FABP3 accumulations.

**Case**	**Disease**	**Age (years)**	**Sex**	**Disease duration (years)**	**Region**	**p-α Syn-ir+ and FABP3-ir+ aggregates**	**α Syn fibril-ir+ and FABP3-ir+ aggregates**
1	PD	75	Male	NS	SN	++	++
					EC	++	++
2	PD	68	Female	10	SN	++	++
					EC	++	+
3	PD	87	Female	9	SN	++	+
					EC	+++	++
4	PD	75	Female	17	SN	++	++
					EC	++	+
5	CN	62	Male	NS	SN	–	–
6	CN	79	Male	NS	SN	–	–
7	CN	83	Female	NS	SN	–	–
8	CN	69	Male	NS	SN	–	–
9	MSA	77	Female	3	ST	+++	+++
10	MSA	62	Male	6	ST	+++	+++
11	MSA	68	Male	7	ST	+++	+++
12	MSA	64	Male	13	ST	+++	+++
13	MSA	76	Female	8	ST	+++	+++
14	MSA	87	Female	23	ST	+++	+++

*PD, Parkinson’s disease; MSA, multiple system atrophy; CN, control subject; SN, substantia nigra; ST, striatum; EC, entorhinal cortex; NS, not specified. Double-positive aggregates in a 20× field: +++, more than 5 intracellular aggregates in a field; ++, 2–5 aggregates; +, one aggregate; –, no aggregates.*

We next examined whether αSyn fibrils were colocalized with FABP3 in synucleinopathies. Similar to the correlation between p-αSyn and FABP3 protein expression in PD and MSA, a strong association between FABP3 protein expression and αSyn fibrils was observed in PD and MSA (Table 1). Figures 2F–J shows images of IF staining of FABP3 (red) and αSyn fibrils (green) in the striatum in MSA tissues (Figure 2F, low magnification; 2G, high magnification) and in the EC in PD tissues (Figure 2H, low magnification). There were no significant differences in the ratio of FABP3 accumulations to αSyn fibrils between PD and MSA tissues (Figure 2J) (*t* = 1.745, *p* = 0.119). FABP3 accumulations were colocalized with approximately 80% of the αSyn fibrils in both PD and MSA tissues (Figure 2J). In contrast, αSyn fibrils were not observed in the SN in CNs, although FABP3 was detected in DA neurons (Figure 2I).

### FABP3 Is Not Expressed in Senile Plaque-Related Amyloid β and Neurofibrillary Tangle (NFT)-Related Phosphorylated Tau (p-Tau) Aggregates in AD

**FIGURE 3:**
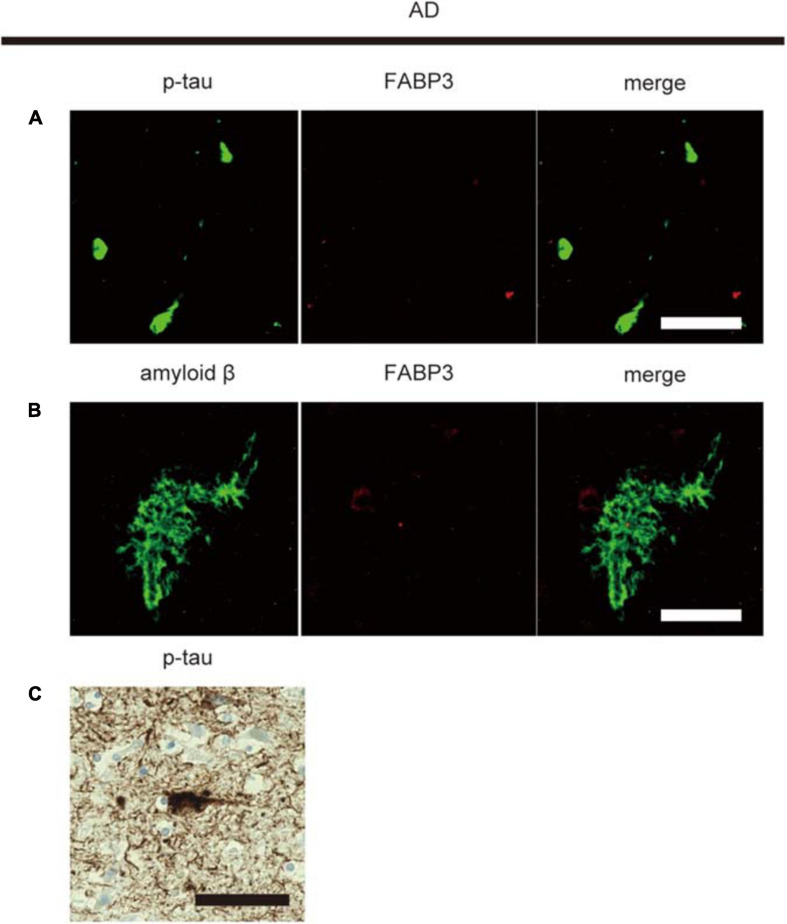
No FABP3 protein expression in AD pathologyIF staining of FABP3 (red) and p-tau aggregates (green) in the frontal cortex in AD tissue **(A)**. IF staining of FABP3 (red) and amyloid β aggregates (green) in the frontal cortex in AD tissue **(B)**. FABP3 accumulation was not observed in amyloid β aggregates **(A)** or p-tau aggregates **(B)** in AD tissue. Nuclear staining with hematoxylin showed that p-tau-positive aggregates were NFTs **(C)**. Scale bars = 50 μm.

Then, we examined whether senile plaque-related amyloid β or NFT-related p-tau aggregates are colocalized with FABP3 in AD. We did not observe FABP3 protein expression in amyloid β-positive senile plaques or p-tau-positive aggregates (Figures 3A,B). Nuclear staining with hematoxylin showed that p-tau-positive aggregates were NFTs (Figure 3C). FABP3 protein in neurons with p-tau-ir aggregates could not be detected in AD. In quantitative analysis, FABP3 accumulations were colocalized with 0% of the senile plaque-related amyloid β-ir aggregates and NFT-related p-tau-ir aggregates in the frontal cortex in AD. Thus, FABP3 accumulation is not associated with senile plaques or NFTs in AD.

### FABP3-ir Accumulations Are Not Detectable in Biopsied Skin Tissues From Patients With PD

**FIGURE 4:**
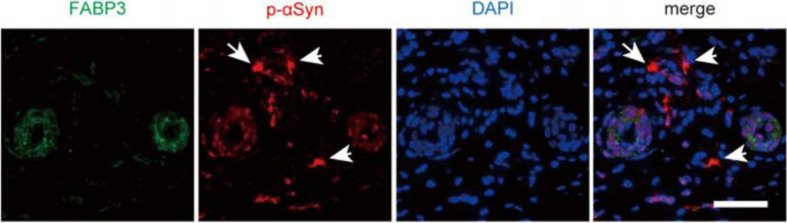
No correlation between p-αSyn deposits and FABP3 accumulation in biopsied skin tissues of patients with PD. IF staining of p-αSyn (red) and FABP3 (green) in biopsied skin tissues of patients with PD. The nuclei were identified by DAPI (blue) staining. Weak FABP3 protein expression was observed in the cytoplasm of dermal sweat duct cells of patients with PD. On the other hand, p-αSyn deposits were observed in the dermal cells (arrows) of patients with PD. FABP3 accumulation was not observed in dermal p-αSyn deposits in PD tissues. Scale bars = 50 μm.

Finally, we examined whether αSyn deposits were colocalized with FABP3 in biopsied skin tissues from individuals with PD. We enrolled relatively early-stage PD patients with normal to mildly impaired cognition for skin biopsy (Table 2). However, dermal FABP3-ir accumulations were not observed in p-αSyn deposits in the samples of any of the patients with PD (Figure 4 and Table 2).

**TABLE 2:** Demographic profiles of PD patients with skin biopsy tissues and the results of immunostaining for FABP3 accumulations and p-αSyn deposits.

**Case**		**Sex**	**Age**	**Duration**	**MMSE**	**FABP3 accumulaions**	**p-aSyn deposits**
1	PD	Female	56	8	29	–	+
2	PD	Male	77	3	27	–	+
3	PD	Male	70	4	26	–	+
4	PD	Female	67	2	27	–	++
5	PD	Female	74	20	27	–	+
6	PD	Male	72	3	23	–	+
7	PD	Male	62	10	30	–	++
8	PD	Male	65	8	29	–	++
‘9	PD	Female	74	8	28	–	+
10	PD	Male	61	2	29	–	++

*PD, Parkinson’s disease; MMSE, Mini-Mental State Examination. Double-positive deposits in a 20× field according to the following scoring system; ++, more than 30 deposits in a field; +, 1–29 deposits in a field; and –, no deposit.*

## Discussion

### Main Results of Our Study

The main results of the present study were that (1) a variety of neurons expressed the FABP3 protein in human brain tissues, (2) FABP3 was colocalized with αSyn aggregates in the brain tissues of patients with synucleinopathies but not with amyloid β or p-tau aggregates in the brain tissues of patients with AD, and (3) FABP3 was not present in p-αSyn deposits in biopsied skin tissues of patients with PD. These findings suggest that FABP3 is associated with αSyn aggregates in the brains of humans with synucleinopathies.

### FABP3 Protein Expression in CNs and Patients With Synucleinopathies

We have previously reported that the FABP3 protein is specifically expressed in DA neurons in brain tissues of wild-type mice (Shioda et al., 2014). We have also previously reported neuron-specific expression of FABP3 mRNA in the rat brain (Owada et al., 1996). Immunohistochemical studies in monkey brains have shown that cerebellar FABP3-positive cells are Purkinje cells and Bergmann glia (Boneva et al., 2010). Thus, FABP3 has been shown to be expressed in mouse, rat and monkey brain neurons. However, whether FABP3 is expressed in human brain tissues has remained unclear. Here, we histologically investigated FABP3 protein expression in autopsied human brain tissues and found that FABP3 was expressed in a variety of neurons, including DA neurons. These data suggest that FABP3 is commonly expressed in mammalian neurons.

We have previously suggested that the FABP3 protein plays crucial roles in αSyn aggregation in an MPTP-induced PD mouse model (Shioda et al., 2014). Long-term oral administration of the FABP3 ligand also significantly improves motor impairments, inhibits MPTP-induced αSyn aggregation, and prevents the loss of DA neurons in the SN (Matsuo et al., 2019). In the present study, we demonstrated, for the first time, that FABP3 colocalizes with αSyn aggregates in the contexts of PD and MSA. In this study, the number of co-localization of FABP3 with p-αSyn aggregates were more observed in MSA compared to PD. We speculated that this was because there was no significant difference in the ratio of FABP3 accumulations to p-αSyn aggregates between PD and MSA, but the number of p-αSyn aggregates were more observed in MSA compared to PD. On the other hand, FABP3 was not associated with amyloid β- and p-tau-positive aggregates in AD tissues. In summary, these results suggest that FABP3 is specifically associated with αSyn aggregates in individuals with synucleinopathies.

Blood FABP3 protein levels in PD were elevated but not in AD, and blood FABP3 protein levels is a potential diagnostic marker for PD (Mollenhauer et al., 2007b; Wada-Isoe et al., 2008b). On the other hand, cerebrospinal fluid FABP3 protein levels were elevated in AD (Guo et al., 2013; Chiasserini et al., 2017). It is reported that blood FABP3 levels were not elevated in AD, but FABP3 expression were observed in astrocyte structures in AD brain (Teunissen et al., 2011). Therefore, FABP3 expression in AD may be involved in the astroglia of the central nervous system. In this study, we analyzed the expression of FABP3 focusing on neurons. Pathological analysis relationship between FABP3 and astroglia should be performed in future studies, because we did not perform the analysis of FABP3 expression in astroglia.

αSyn aggregation is well recognized to contribute to the pathogenesis of synucleinopathies. Whereas only a small fraction of αSyn (<4%) is phosphorylated in healthy brains, distinct accumulation of αSyn phosphorylated at S129 (pS129) (>90%) is observed in LBs and GCIs (Oueslati, 2016). LBs and GCIs are composed of insoluble fibrillar protein aggregates and are responsible for cell death in synucleinopathies (Oueslati, 2016). Conversion of αSyn from a soluble monomer into oligomeric species and insoluble fibrils underlies the neurodegeneration associated with PD (Kalia et al., 2013). Previous reports have suggested that αSyn binds to FAs, particularly long-chain polyunsaturated fatty acids (Perrin et al., 2001; Sharon et al., 2001). Exposure to FAs enhances αSyn aggregation in cultured mesencephalic neuronal cells (Sharon et al., 2003; Liu et al., 2008). αSyn also binds to FABP3, and αSyn aggregates with FABP3 accumulation are detectable in damaged DA neurons in MPTP-induced PD model mice (Shioda et al., 2014). Therefore, αSyn aggregates and FABP3 may interact with each other and play a key role in the pathogeneses of synucleinopathies.

Recently, dermal αSyn deposits in biopsied skin tissue have been proposed to be useful biomarkers for the diagnosis of relatively early-stage PD (Ikemura et al., 2008; Donadio et al., 2014; Zange et al., 2015). Here, we histologically investigated FABP3 protein expression in biopsied skin tissues of patients with PD. However, FABP3 accumulation was not observed in p-αSyn deposits in these tissues. Generally, FABP3 is highly expressed in mature neurons in the central nervous system (Boneva et al., 2011). In the FABP family, myelin FABP (FABP8) is expressed in the peripheral nerves, and brain FABP (FABP7) and epidermal FABP (FABP5) are expressed mainly in glial cells and neurons of the immature brain (Boneva et al., 2011). Thus, p-αSyn deposits might colocalize with other FABPs (e.g., FABP5, FABP7 and FABP8) in PD skin. Further histological analyses of other FABPs (e.g., FABP5, FABP7 and FABP8) in PD skin should be performed in future studies.

## Conclusion

In the present study, we demonstrated, for the first time, that FABP3 is associated with αSyn aggregates in autopsied brain tissues of patients with synucleinopathies but not with amyloid β or p-tau aggregates. These results provide new insights into the involvement of FABP3 in synucleinopathies.

## Data Availability Statement

The original contributions presented in the study are included in the article/supplementary material, further inquiries can be directed to the corresponding author/s.

## Ethics Statement

The studies involving human participants were reviewed and approved by Sendai Nishitaga Hospital Local Human Ethical Committee. The patients/participants provided their written informed consent to participate in this study.

## Author Contributions

HO, KY, HS, TH, KF, and AT designed the study. HO, KY, HS, and YS performed the experiments. HO, KY, and HS analyzed the data. HO, KY, HS, TB, and AT wrote the manuscript. All authors contributed to the article and approved the submitted version.

## Conflict of Interest

The authors declare that the research was conducted in the absence of any commercial or financial relationships that could be construed as a potential conflict of interest.

## Abbreviations

αSyn: α-synuclein
FABP: fatty acid-binding protein
DA: dopaminergic
PD: Parkinson’s disease
CN: control
SN: substantia nigra
LBs: Lewy bodies
MSA: multiple system atrophy
GCIs: glial cytoplasmic inclusions
NCIs: neuronal cytoplasmic inclusions
DLB: dementia with Lewy bodies
MPTP: 1-methyl-1,2,3,6-tetrahydropyridine
FAs: fatty acids
p- α Syn: phosphorylated α Syn
ir: immunoreactive
p-tau: phosphorylated tau
NFT: neurofibrillary tangle
PUFAs: long-chain polyunsaturated fatty acids
AD: Alzheimer disease.
